# The impact of COVID‐19 on franchising in emerging markets: An example from Brazil

**DOI:** 10.1002/joe.22053

**Published:** 2020-06-22

**Authors:** Vanessa Pilla Galetti Bretas, Ilan Alon

## Abstract

The outbreak of COVID‐19, the disease caused by the SARS‐CoV‐2 virus, has had significant economic, political, and social consequences worldwide. The franchising sector, consisting mostly of retail and service businesses, is an example of an industry that has been deeply affected. The experiences of franchising stakeholders in Brazil highlight the strengths of the franchising model in such situations. This investigation, based on primary data from webinars with food service, education, retail, and business‐to‐business service companies in Brazil, coupled with reports from commercial and franchising entities, reveals how the COVID‐19 outbreak has affected the franchising sector. It illustrates the measures that were taken, the negotiations that take place between suppliers and landlords, the adaptation of business models, the effects on franchisor‐franchisee relationships, and the impact the pandemic has had on relationships with customers. The strategies adopted by Brazilian franchisors and franchisees suggest lessons for other franchising companies in similar situations, such as those in developing and emerging economies.

## 1 INTRODUCTION

The COVID‐19 pandemic is one of the greatest challenges the modern world has faced. The disease, caused by the severe acute respiratory syndrome coronavirus 2 (SARS‐CoV‐2), can be traced back to December 2019, when the first case was reported in Wuhan, China. In March 2020, the World Health Organization declared a pandemic, meaning that the disease was spreading worldwide. In order to slow down the infections and “flatten the curve” of the epidemic—that is, reduce the rate of transmission—several countries have suspended business activities, and adopted social distancing to reduce person‐to‐person contact. Governments are struggling to simultaneously save lives, and mitigate the economic impact of the virus (Anderson, Heesterbeek, Klinkenberg, & Hollingsworth, 2020; Rodriguez‐Morales et al., 2020; Surico & Galeotti, 2020).

Numerous uncertainties surround the disease. Many of the characteristics of COVID‐19, such as the duration of the infectious period, symptomatology, and the possibility of asymptomatic transmission, are either speculative or unknown. The development of a vaccine or possible treatment is a long‐term project, which might take years to accomplish. Thus, social distancing measures such as isolation, the banning of mass gatherings, and the closing of schools and stores are, at present, the most effective ways to reduce the rate of transmission, avoid the collapse of healthcare systems, and minimize the number of deaths (Anderson et al., 2020).

## 2 COVID‐19 IN EMERGING ECONOMIES

COVID‐19 has had a damaging effect on the economy of countries around the world, but perhaps more particularly so in emerging economies. Although emerging economies are heterogeneous, they do share some common characteristics, such as weaker institutional and legal settings, lower levels of economic development, and higher levels of financial and social risk (Hevia & Neumeyer, 2020; Surico & Galeotti, 2020). These challenging economic and institutional conditions limit the range of their responses to the COVID‐19 outbreak. Governments have fewer alternatives with which to confront the pandemic, guarantee health care, offer adequate social protection, and deal with the economic consequences of a pandemic (Buchanan, Anwar, & Tran, 2013; Cuervo‐Cazurra, 2012; Ramamurti, 2012; Stiglitz, 2020).

Besides the devastating impact of the pandemic on the weak health infrastructure in these countries, the need for prolonged social distancing and other mitigation policies have led to an economic slump, which in turn has had adverse effects on levels of unemployment. Moreover, most emerging economies are dependent on exports; they are also affected by the contraction of international trade, the fall of commodity prices, currency devaluations, and disruptions to the global supply chain. A report from the United Nations Conference on Trade and Development (UNCTAD, 2020) projects that, due to the pandemic, developing countries (excluding China) will lose USD 800 billion in export revenue in 2020.

Furthermore, these countries have other constraints that make it harder for them to take the necessary measures. Many jobs are informal and consist of the type of work that cannot be done from home. Most have poor housing and sanitary conditions, where people live in close proximity, making social distancing difficult or impossible. Their economies are characterized by high levels of social inequality, which contributes to the risk of further disruption. Control measures that require the suspension of business activities and social isolation have very different consequences in a developed economy, where the workforce might expect access to free healthcare and income protection, than in an economy where people have to choose between starvation or going to work and risking their health.

Throughout the world, COVID‐19 has presented governments with a choice between preserving lives and the capacity of the health care system to deal with the pandemic, and the financing of policies to mitigate the costs of social distancing and shuttering businesses. However, it is important to recognize that the implementation of such measures is significantly more challenging for an emerging economy (Hevia & Neumeyer, 2020; Lemos, Almeida‐Filho, & Firmo, 2020; Stiglitz, 2020).

## 3 EFFECTS ON THE FRANCHISING SECTOR

Franchising as a commercial and social model has several economic and social effects, such as job creation, economic modernization, and the development of entrepreneurship (Alon, 2004; Naatu & Alon, 2019). The direct impacts on income, employment, and the achievement of social goals are most noticeable in emerging and developing markets (Alon, Welsh, & Falbe, 2010; Elango, 2019; Naatu & Alon, 2019).

According to the World Franchise Council’s 2017 survey on the economic impact of franchising worldwide, India, Taiwan, and Brazil ranks among the top five countries worldwide in the number of franchise brands (**Exhibit** **1**). South Africa is the country with the second highest share of the country’s overall GDP generated by the franchising sector, 15.3%. Brazil ranks fifth in terms of job creation by the franchising sector, employing almost 1.2 million people in 2017 (ABF, 2020; FASA, 2020a).

**EXHIBIT 1:**
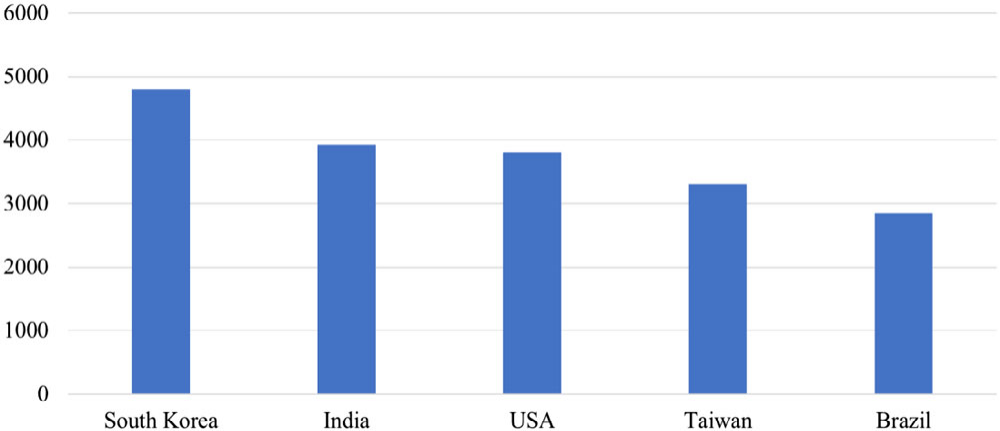
Top five countries by number of franchise brands [Color figure can be viewed at wileyonlinelibrary.com]

Because of the size of their population, per capita income, urbanization rates, and income distribution, emerging markets offer the largest and most dynamic markets for international franchisors. Emerging economies have used global franchising as a tool for economic and entrepreneurial development, job creation, and global integration (Alon, 2004, 2006; Alon & Lattemann, 2016; Alon, Toncar, & McKee, 2000; Baena, 2012; Welsh & Alon, 2001).

The negative effects of the pandemic on the economies of developing and emerging countries are worrying. One of the first consequences of the crisis has been the withdrawal of investment from countries considered to be at greatest risk. Countries that depend on exports of commodities or manufacturing goods, such as China, Mexico, and Brazil, are suffering from a drop in demand. Furthermore, tourism, which is an essential source of revenue for many developing and emerging countries, is paralyzed (Fariza, 2020; Stiglitz, 2020).

The effects of the COVID‐19 outbreak on other services and retail industries are also severe. Construction, food service, fashion, and retail are some of the sectors most affected by the pandemic. The franchising sector is also strongly impacted, with consequences for the business activities and integrity of the franchise system. The crisis also creates additional challenges to the dynamic between franchisors and franchisees.

Although mostly related to services and retail, franchising covers a broad spectrum of business activities, and is affected by the crisis to different degrees (Abell, 2020; Sebrae, 2020; Surico & Galeotti, 2020; Teixeira, 2020). A study (Teixeira, 2020) by FIAF, the Ibero‐American Franchising Federation, which represents the franchise associations of Portugal, Mexico, Guatemala, Costa Rica, Panama, Colombia, Venezuela, Ecuador, Peru, Brazil, Uruguay, Paraguay, and Argentina, showed that retail franchisors are the most affected by the coronavirus crisis, followed by food service franchises (**Exhibit** **2**).

**EXHIBIT 2:**
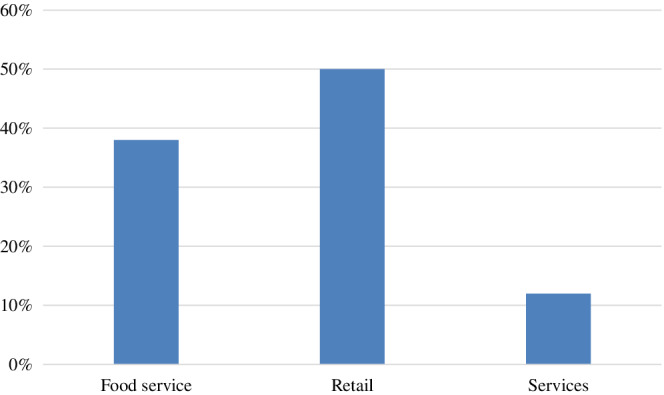
Most affected franchisors in the Ibero‐American franchising sector [Color figure can be viewed at wileyonlinelibrary.com]

In this context, the impact of COVID‐19 on the BRICS nations—Brazil, Russia, India, China, and South Africa—are of concern because they account for a large share of the global GDP and population. The social distancing measures are severely impacting the franchising, retail, and services sectors in these countries. In South Africa, since the implementation of the COVID‐19 emergency policies, approximately 94% of the franchising industry has not been operating. The lockdown measures included food deliveries. The Franchising Association of South Africa made a public statement asking for the takeout and fast food sectors to be included as essential services (FASA, 2020b).

The joint report from the China Chain Store & Franchise Association and Deloitte China (Deloitte, 2020a) shows that retail businesses are facing several operational and financial challenges in the country. Among the companies surveyed, 90% said that the pandemic had impacted the number of customers, especially stores and food service chains operating in shopping centers.

The Retailers’ Association of India (2020) surveyed retail companies to understand the impact of COVID‐19 on their businesses. More than 95% of nonfood retailers have had their stores closed. Food retailers expect to earn only 56% of last year’s revenues. The country is facing a massive decline in restaurant and food service businesses. Deliveries have become the primary source of income for these companies (Deloitte, 2020b). In Russia, all retail stores are closed, except for food stores and stores providing essential goods. Restaurants are open only for takeaways (Dentons, 2020).

## 4 THE IMPACT OF COVID‐19 IN BRAZIL

In Brazil, the first confirmed case of COVID‐19 was a 61 year‐old man, who had been traveling in Lombardy, Italy, and arrived in São Paulo on February 21, 2020. The Brazilian Ministry of Health confirmed the case on February 26, 2020. On March 13, the state health departments across the country announced recommendations to limit the spread of the disease. The government recognized community transmission across the country on March 20 (Croda et al., 2020; Rodriguez‐Morales et al., 2020).

To try to reduce the number of cases and flatten the pandemic curve, the Brazilian health authorities reacted by implementing measures such as isolation, quarantine, and temporary restrictions on entering and leaving the country. Despite this, the country still faces some major challenges when trying to alleviate the worst effects of the pandemic (Croda et al., 2020; Surico & Galeotti, 2020).

At the end of February, when the first case of COVID‐19 was confirmed, the projections for the growth of Brazil’s GDP in 2020 were around 2%, already signaling a weak expansion (Banco Central do Brasil, 2020). The coronavirus outbreak generated a supply and demand crisis, and will have a strong negative impact on the Brazilian GDP, resulting in unemployment and income losses. More than 40% of Brazil’s workforce is employed in the informal sector (IBGE, 2020). Consequently, the effects of prolonged quarantine are devastating (Surico & Galeotti, 2020).

Moreover, like other emerging economies, Brazil is characterized by sizeable social inequality. It is a country the size of some continents, with an estimated population of 209 million in 2018; it also has the most populous city in South America, São Paulo, with a population of more than 21 million people in the greater metropolitan area. The wealthiest part of the country, around the capital Brasília, has a GDP per person equivalent to Italy, while the most impoverished region, the States of Maranhão and Piauí, have a GDP per capita comparable to Jordan (Economist, 2014; Rodriguez‐Morales et al., 2020).

A large part of the population lives in deplorable conditions in the cities’ outskirts or “favelas,” where, in addition to poor healthcare and sanitation, overcrowding is such that it is almost impossible to implement social distancing. According to a study developed by the Trata Brasil Institute, 16.38% of the Brazilian population does not have access to a clean water supply, and only 46% of the volume of sewage generated in the country is treated. Since hygiene is one of the most important measures against the spread of the disease, these conditions impose severe limitations on the ability of many people to take preventive actions (Instituto Trata Brasil, 2020; Macedo, Ornellas, & do Bomfim, 2020).

As in some other emerging economies, such as Africa, South Asia, and Latin America, the capacity of the Brazilian health care system to deal with a pandemic is severely limited. The Brazilian population consists mainly of young adults with a high incidence of diseases such as diabetes, hypertension, obesity, HIV, and tuberculosis. The country has a universal health care system; however, in terms of the availability of intensive care units, equipment, and diagnostic tests, the outbreak of COVID‐19 has placed additional pressure on an already vulnerable system (Croda et al., 2020; Surico & Galeotti, 2020).

In response to these challenges, the Brazilian government has adopted some measures to mitigate the impact of the crisis on the economy. The main provisions include direct and indirect tax measures, employment‐related measures, and an economic stimulus (KPMG Global, 2020).

- Tax measures

The indirect and direct tax measures include payment deferrals and rate reductions. The economic stimulus package announced on March 26 includes funds to replace the extension or suspension of the payment of taxes, a Guarantee Fund (FGTS), and a reduction in contributions.

- Employment‐related measures

Several provisional measures were enacted to adapt labor regulations during the pandemic, and to provide aid to employers, employees, informal workers, and autonomous social services. These measures include the possibility of working remotely, provision for individual and collective vacations, and compensation for working unusual hours. Other provisions include the payment of job and income preservation benefits, permitting a proportional reduction of working hours and wages, and the temporary suspension of employment contracts. Emergency aid to informal workers and a reduction to the rate of contributions by autonomous social services were also granted.

- Economic stimulus measures

Among the measures to stimulate the economy are credit lines and loans for micro, small, and medium‐sized companies, a simplification of the requirements to obtain credit, flexibility in the rules for obtaining loans, a moratorium on debt repayments, and credit lines for small and medium‐sized companies to pay salaries. A list of all of the measures adopted so far appears in “Brazil’s policy responses to COVID‐19” published by the Secretariat for International Economic Affairs (Ministério da Economia, 2020).

## 5 THE RESPONSE OF BRAZIL’S FRANCHISING STAKEHOLDERS

In 2019, the Brazilian franchising sector’s revenue was USD 46.3 billion, with 2,918 franchisors operating 160,958 establishments in the country. Brazilian franchisors were responsible for 1.36 million direct jobs in the country (Brazilian Franchising Association [ABF], 2019). A recession in this sector has a significant negative impact on the Brazilian economy in terms of employment and revenue losses.

The ABF, which represents the industry’s stakeholders and has contributed to their best practices since 1987, took several measures to minimize the impact of this crisis on the franchising system. It is developing advocacy initiatives with the federal and state‐level governments, and with banks and other associations. Another effort centers on webinars and online roundtable discussions with franchisors, franchisees, and other relevant players.

Three webinars and five roundtable discussions were conducted online from April 7 to April 27; the participants included 17 franchisors, 7 franchisees, and 5 consultants. The discussions focused the strategies being adopted by companies, and gaining insights for the future. The franchisors and franchisees that participated represented the food service, education, retail, and business‐to‐business service sectors.

In the following sections, we review the main ideas discussed regarding five topics: initial measures adopted, negotiations with suppliers and landlords, business model adaptations, the franchisor–franchisee relationship, and the relationship with customers. Some direct quotations of the participants’ opinions are provided to illustrate certain points.

### 5.1 The first measures were emergency care

Both franchisors and franchisees had to close the doors of their stores at the end of March. In general, the first measures they took were revising their budgets and dealing with their employees. A primary concern was to find ways to preserve their cash flow, by reducing costs, and by maintaining at least part of their revenue through alternative channels.

The franchise chains started by reducing or eliminating all nonessential expenses such as consulting services, communication services, store repairs, and maintenance. Several companies decided to terminate temporary and work‐experience contracts, while permanent employees were encouraged to take their vacation days or to work remotely. After the isolation measures were introduced, the franchising firms began a temporary suspension of employment contracts and a reduction in work hours and wages. Both franchisors and franchisees regarded layoffs as a last resort.

At the same time, franchisors and franchisees started to apply for credit lines to finance salaries. As part of the Brazilian government’s economic stimulus measures, private banks, serving as intermediaries between the public banks and enterprises, started offering credit. However, several companies reported difficulty in accessing these credit lines due to differences between the public and private sector banks about how to manage the process. The ABF began negotiating with the banks to release working capital lines for franchisees.

There is a general perception that these were initial strategies that needed to be revised continuously. One franchisee stated, “We are living in the moment. After 30 days, we must revisit some decisions that we made. There is great uncertainty regarding our decisions since the paths are not very clear.”

### 5.2 Relations with all of the players are becoming increasingly close

Negotiations with suppliers and commercial property landlords are essential to guarantee financial breathing room for franchisors and franchisees. These negotiations are complicated because all of the parties involved are trying to reduce expenses and maintain at least part of their income. The suppliers of franchise chains are also strongly affected by the crisis. The shopping malls depend on rents and fees. Some of the street stores’ landlords are individuals or family owners for whom rent is their primary income. A franchisee of a food service chain commented, “Many are family properties, many people who depend on that income, that rent.”

In this context, the risk that negotiations will end in a lose–lose situation is high. Thus, despite specificities of each negotiation process, because every stakeholder is facing an extraordinary situation, a shared vision has emerged among franchising companies of the importance of flexibility. Another franchisee stated, “We are looking for a negotiation with all our partners, service providers, and suppliers where the two parties lose less.” Being part of a franchise is an advantage in the negotiations because of the benefits of scale; it is better to negotiate as part of a network than to negotiate individually.

Several shopping malls are allowing food operations to open for a delivery‐only service and offering to delay, discount, or cancel the payment of rent for March and April. Other suppliers are negotiating the postponement or a temporary suspension of payments. However, according to the franchisees, most of the suppliers and landlords have not put forward any alternatives for the long term. Franchising companies are concerned about how to renegotiate their contracts and manage the return to business after COVID‐19. The franchisee of a cosmetic company noted, “We are looking forward to more transparent negotiations when the moment for activities return. The criteria that are in our contracts now will not enable our recovery.”

The ABF asked Abrasce, the association representing the shopping malls, for measures to cover the immediate future. Some of the suggested measures include the payment of proportional rent until the end of 2020, exemption from the advertising fund, proportional deductions from the condominium, discounts related to opening hours, and the elimination of transfer fees for 2020.

### 5.3 It was necessary, but now it is inevitable

Franchisors and franchisees have begun implementing adaptations to their business models to lessen the effects of the crisis. Many developments that were seen as necessary in the medium or long term are considered inevitable now. The most significant change is the speed with which franchisors and franchisees have embraced technology. The crisis has intensified already‐existing trends related to consumer behavior, organizational structure, and the supply chain, and have affected every part of the businesses. Processes and technologies that were previously seen as experimental, such as digitalization, online purchasing, mobile technology, and omnichannel marketing, are now being more widely implemented due to the challenges posed by the pandemic.

In the food service sector, some restaurants are offering delivery for the first time and the delivery model is seen as one that is likely to continue. Other chains that had been working with delivery before are now increasing their investment in this model. A franchisee of a food service chain said, “We are specializing in delivery using a lot of [different types of] technology.” Retail franchise companies are also investing in delivery, using marketplaces as a sales channel, or developing their own delivery structure.

Another model being discussed by firms in the food sector is the “dark kitchen” model, where meals are prepared to order and exclusively for delivery. According to a franchisee of a food company that sells açaí “We have a plan to open a dark kitchen that operates only on delivery. The occupancy cost decreases, with reduced operation hours.” However, some companies believe that the revenue from delivery sales will begin to diminish as ordering food is a nonessential expense for a significant part of the Brazilian population, especially with the expected growth of unemployment.

In the education sector, which includes second‐language schools, academic tutoring, and training institutes, franchise chains are rapidly developing online classes. Most schools will offer payment renegotiation, or the possibility of postponing courses, to students who do not feel comfortable having online courses. Franchisors consider online courses to be a temporary measure, claiming that students prefer face‐to‐face interaction. For example, the franchisor of a second‐language school said “Our intention, as soon as possible, is to return to traditional classes. We believe that face‐to‐face courses will rarely be replaced.” However, the move toward an online environment is a source of concern for some franchisees. The online platform becomes a direct channel between the franchisor and the final consumer, which could impact on the role of the franchisee after the crisis.

In sectors such as retail, companies are also adopting digital tools, and e‐commerce sales are increasing. In 2019, 61% of the franchisors operating in Brazil used e‐commerce as a sales channel. This figure will undoubtedly rise in 2020. A strategy adopted by some franchising companies is “future sales”: customers buy products with discounts now and take them later when the stores reopen. In addition, both franchisors and franchisees have started using tools such as WhatsApp and Instagram for sales, and are developing virtual catalogs and adapting their distribution, delivery, and payment routines. According to a franchisee of a beauty company, “WhatsApp is a tool for selling that is very efficient, very agile.”

### 5.4 Building bridges between franchisors and franchisees

The structure of the franchise model facilitates the exchange of information and ideas which can help to reduce the negative impact of the crisis. The collaboration between franchisors and franchisees allows them to react more quickly and make decisions with greater certainty. The franchisor offers financial and managerial support to the franchisees. On the other hand, the franchisees help the franchisors identify threats and opportunities for the network. The words of the president of the ABF highlight how the franchising system in Brazil is dealing with the crisis: “Franchisors and franchisees are working together, being flexible and adapting rules, models, concepts.”

In general, franchisors are renegotiating or suspending the payment of royalties and fees. Several franchise chains have created crisis committees that include franchisees and members of different areas of the franchisor to exchange ideas and discuss measures. The crisis committees accelerate processes. In addition, franchisors are investing in communication with the franchisees. They share information about their business and other topics, such as health, personal care, negotiation, and legal advice, through webinars, live discussions, and podcasts.

For instance, a franchising group that includes accessories and fashion brands implemented a three‐phase process with the franchisees. The first phase consisted of postponing or renegotiating the payments of royalties and fees. The next step was the analysis of the franchisees’ financial situation and the need for working capital. And the last one consisted of individual guidance for each franchisee.

A second‐language school devised a plan to offer online classes in 3 weeks. During the development of the strategy, the franchisor discovered that one franchisee already offered online courses. In this way, the network was able to shorten the path by learning from what the franchisee had done and replicating it with other franchisees.

So far, the franchisees’ overall perception of the franchisors’ actions regarding the pandemic is positive. “There is a very healthy exchange between franchisor and franchisee to redesign the business.” They asserted that most of the franchisors had opened communication channels, were negotiating measures, and were trying to understand the franchisees’ needs. Franchisors are helping in the negotiations with suppliers and landlords. Some of them are giving franchisees legal advice and commercial support.

### 5.5 The customer’s behavior will change; our behavior will change

Franchising firms believe that during the crisis and the recovery period, customers will be primarily concerned with basic necessities. A second‐language school franchisee asserted: “We have a considerable challenge because customers will prioritize [goods and services] that will meet their basic needs.” Consumer confidence is eroding. Due to large numbers of informal workers without an income, the suspension of employment contracts, and an unemployment rate that will continue to rise, a substantial part of the Brazilian population is cutting out all nonessential expenses. According to Instituto Brasileiro de Economia (FGV‐IBRE, 2020), 80% of Brazilian consumers are only buying essential goods, such as food and health care products; consequently, franchise chains in sectors that offer goods or services that are considered superfluous are facing enormous difficulties.

Most franchisors and franchisees are intensifying communication with their customers, trying to get closer, and to maintain their relationship. The franchisor of an accessories brand decided not to focus on sales at this moment, understanding that their customers have other priorities, “Our communications are concentrated on embracing our customers, to say that we are together right now.” Franchisors in the education sector are also investing in maintaining communication with the students, offering special payment plans to keep enrollments. Franchise chains in business‐to‐business industries are adjusting their marketing campaigns, to target those clients who are in sectors where there is still a demand.

Currently, changes in the amount of face‐to‐face interaction mean that businesses have shifted toward online shopping and delivery, investments in omnichannel marketing, and the digitalization of sales. However, in the longer term, other aspects related to the interaction with customers are expected to emerge. For example, companies will need to encourage stores to accept digital payments, guarantee hygiene measures, rethink the provision of food, and experiment with new ideas for clothing outlets. A retail franchisor that sells glazed roasted nuts is revising several of its processes for the recovery period: “We will have to change our tasting process, the product display, and the packaging assembly. Our idea is to show customers everything we do, so the customer will be sure that the product is made in a safe way, and that he or she is not taking any risks.”

Another unanswered question about the consequences of the pandemic is how this crisis will affect consumer behavior in the long term. As a result of the health crisis, will companies have to deal with a more environmentally conscious consumer; one that is more concerned with sustainability and social issues? Will preexisting trends such as a reduction in consumption, the change from ownership to rental services, and the repurposing of items be intensified? Stakeholders in the franchising sector believe that consumer behavior will inevitably change, and that companies will have to change too. The comments by the head of the ABF digital transformation committee highlight this perception, “The crisis is making people think about what they are consuming, and why they are consuming it.”

## 6 BEST PRACTICES AND THE IMPLICATIONS FOR FRANCHISING COMPANIES

The experiences of the Brazilian franchising stakeholders, from food services, education, retail, and business‐to‐business services, offer a number of lessons to other companies facing the same challenges in similar contexts. A synthesis of their best practices appears in **Exhibit** **3**.

- Several franchisors created multidisciplinary crisis committees, which included a number of different franchisees, to discuss alternatives, establish necessary measures, and create strategies. Having these committees accelerated the speed with which decisions could be taken.
- A thorough evaluation of the company’s budget is crucial. The main concern for franchisors and franchisees, from all business areas, was to preserve their cash flow by reducing expenses and maintaining at least part of their revenue. Nonessential and temporary contracts were reviewed or canceled. Employee‐related measures such as salary reductions and the suspension of employment contracts were widely adopted.
- Franchising firms also had to make use of government emergency aid, such as credit lines to pay employees’ salaries and cover fixed costs. To achieve this, franchisors and franchisees needed to understand what aid packages were available and how to apply for them.
- Commercial and sectorial associations play an important role in communicating the needs of companies to government and other public entities. They can also help in the negotiation with other players, such as banks and other associations.
- Companies need to negotiate with suppliers and landlords to prevent avoidable losses, by both parties, by reducing fees, offering discounts on rent, and postponing payments.
- To adapt to and exploit changing circumstances, franchising companies need to modify their existing business model, for example, by embracing technology, operating dark kitchens, or offering future sales.
- Franchisors need to help franchisees find appropriate financial responses to the crisis. The opening of new communication channels, such as webinars, podcasts, and multi‐stakeholder online “round‐table” meetings, are one way to achieve this; flexibility in the payment of fees will also help to reduce the immediate pressure on franchisees.
- Communication and transparency with employees is essential. Franchisors and franchisees need to help employees adapt to new modes of working by providing information about what is happening to the business, and about health and welfare.
- Franchisors and franchisees should adapt and intensify their communication with customers by, for example, the use of online tools.
- Much remains unknown about the characteristics of COVID‐19, and new scenarios emerge daily; it is therefore essential that business strategies are reviewed periodically to take account of changes in circumstances.

**EXHIBIT 3:** Best practices from Brazil

Create a multidisciplinary crisis committee
Evaluate the budget, preserve cash flow where possible, and cut nonessential expenses
Understand the emergency aid packages available
Connect with relevant commercial and sectorial associations
Contact suppliers and landlords to renegotiate contracts to reduce losses
Modify existing business models to maintain revenue
Invest in and build upon franchisor–franchisee relationship
Invest in communication and transparency with employees
Adapt and improve of communication with clients
The situation can change quickly; it is necessary to regularly reassess strategies

To summarize, each business sector has its own particular characteristics, will suffer different effects from the crisis, and will need to take its own distinctive actions in response. However, looking at the experience of Brazil, three broad strategies stand out.

The first is the acceleration of digital transformation. Franchisors and franchisees, from all business sectors, have increased the speed with which they have adopted technology to run their business. Trends that were viewed as experimental before the crisis, such as online sales, omnichannel marketing, and mobile technology, have now become essential components for a businesses’ continued operation.

Second, collaboration and communication has become more widespread, and relationships have become more horizontal than vertical. Teamwork between franchisor and franchisees has become a central theme for the franchising sector. One example of this is the creation of crisis committees by franchise chains to consider alternatives to the challenges created by the COVID‐19 outbreak. A similar trend can be seen inside companies, with the relationships between managers and employees becoming more horizontal too.

The third strategy concerns flexibility. The situation created by the pandemic requires stakeholders to be more proactive and adaptable. In addition to the need to adapt business models, flexibility is essential in consultations between franchisors and franchisees, negotiations with suppliers and landlords, and in dialog with employees and customers.

These three strategies—embracing technology, collaboration, and flexibility—are vital for companies trying to keep up with the current changes in the business environment, and for dealing with whatever may come in the future. It remains unclear how long the crisis will last, and what its eventual impact will be; however, as we have seen, stakeholders in Brazil have been able to use the strengths of the franchising model to respond to the challenges. We believe their experiences will be of value to other franchising companies in similar situations.
